# Mapping Privacy Vulnerabilities in Local Area Network (LAN) Environments

**DOI:** 10.3390/s26030763

**Published:** 2026-01-23

**Authors:** Zohar Fine, Ron S. Hirschprung

**Affiliations:** Department of Industrial Engineering and Management, Faculty of Engineering, Ariel University, Ariel 4070000, Israel

**Keywords:** vulnerabilities, communication-networks, LAN

## Abstract

Privacy is a major concern in the digital era and is intensively addressed in academic research, in industry, and by regulators. However, almost all references to privacy in the digital world relate to the Wide Area Network (WAN) environment, which is actually the Internet, whereas the Local Area Network (LAN) environment is neglected. While the Internet is widespread, almost every connection to the Internet is via a LAN. Given the increased interest in privacy, and the popularity of LANs, privacy threats on a LAN should have been extensively addressed. Nonetheless, significant research on LAN privacy issues is limited. Therefore, the focus of this study is on privacy vulnerabilities in the LAN environment. By conducting a literature meta-analysis and, particularly, by interviewing LAN managers and experts, we identified 18 vulnerabilities that may introduce privacy threats. The privacy risk assessment of the vulnerabilities was based on the FMEA approach. In an empirical study, we evaluated these vulnerabilities on 13 different LANs. Excluding one vulnerability, all the others were found on at least one LAN, and more than 50 percent of the vulnerabilities were identified as high-risk. The results show that the LAN is indeed a source of significant privacy concerns.

## 1. Introduction

The digital era affects almost all aspects of our lives, e.g., shopping on e-commerce platforms, consuming media by means of streaming, socializing on online social networks, managing health care and information digitally. These platforms, while beneficial, also introduce significant privacy risks [1]. In fact, the increased use of digital systems also increases the concerns of harming privacy [2]. This phenomenon can be inspected in most Internet services. For example, when uploading pictures to online social networking information is shared, the user’s location may be disclosed, and the people that appear in the picture may also be identified. In addition, when visiting e-commerce stores, making wish lists, or shopping, the preferences of the user are revealed. Usually, private information is used to establish a consumer’s profile, but it can also be used for cyber-attacks or identity thefts [3].

In the European Union, approximately 75% of the digital consumers agree that disclosing personal information is part of modern life and that some privacy loss is an inherent “price” of enjoying “free” services. Others are simply unaware of the tradeoff between exposing their own personal information and receiving “free” services [4]. Privacy has become a significant issue, and privacy protection is addressed by almost every government and regulator throughout the Western world. In the 1970s, the US Department of Health, Education and Welfare had already developed a privacy framework of Fair Information Practice Principles (FIPP). Other governmental institutions, such as the Federal Trade Commission Department of Health and later the Organization for European Economic Co-operation (OECD), also adopted privacy guidelines [5]. In May 2018, the European Union published the General Data Protection Regulation (GDPR), which aspires to protect the privacy of all EU citizens [6,7].

The digital era is based on communication networks, which have the capability to exchange data between two or more devices or systems through a medium such as electrical cables, optical fibers, or wireless connections. This communication allows devices to share information and resources between the devices [8]. There are a few ways to classify networks, e.g., the area (deployment) of the network, speed of communication, types of hardware equipment, and the media carrier, which introduce categories like WAN (Wide Area Network) that is spread between buildings, cities, and countries, and LAN (Local Area Network) that is spread throughout the campus or in one building. Though LAN and WAN are probably the most prevalent types, another way to classify a network is the type of the media carrier, e.g., coaxial cable, twisted pair, fiber optic, and radio wave communication [9].

A Local Area Network (LAN) is a network that connects computers and other devices in relatively small areas, several rooms on a floor in a building, several floors, or a few buildings on the same campus. According to Amazon, “A local area network (LAN) connects devices that are physically close to each other by using connectors like routers and switches. A LAN is usually a private network under private ownership and is used to share resources like printers, storage, and Internet connections (gateways).” Other LAN uses may include data sharing between users that need access to the same files, e.g., by means of a LAN server [10,11]. The LAN connection can be based on wire or wireless media. A wired connection requires specific hardware which contains a channel port that connects all the wires, e.g., a hub that directs the communication to all ports or a switch that directs the communication to a specific port [12]. This network is usually based on Ethernet architecture defined in accordance with the IEEE 802.3 standard [13].

The wireless LAN (WLAN) requires an access point that covers the desired area and a wireless network device for the hosts (the end stations). This connection is usually based on a Wi-Fi architecture defined in accordance with the IEEE 802.11 standard [14]. The Wireless LAN has been widely deployed in private and public places, with some of them free for use. Mobility, flexibility, scalability, cost-effectiveness, and rapid deployment are key factors driving the widespread adoption of this technology. A LAN, whether wired or wireless, is a closed network in which communication is enabled only among hosts connected to the same LAN [15].

The Wide Area Network (WAN), on the other hand, as its name implies, is a network structure that interconnects geographically dispersed locations. This network uses public and private routers that can cross cities, countries, states, and even continents [9]. According to Amazon, “A wide area network spans beyond a single building or large campus to connect multiple locations spread across a specific geographic area or even the world. Organizations use WANs to facilitate digital interactions and data sharing between employees and customers in different regions or countries.” [16]. WAN provides the ability to connect distant networks, e.g., corporate branch offices, thereby enabling employees to remotely work on their office computers and allowing customers or suppliers to connect to the corporate database [17]. The most common and practically almost the only implementation of WAN is the Internet network that connects smaller networks worldwide and enables a variety of services like web browsing, email, streaming, e-commerce, online social networks, and digital news [18].

The Internet is everywhere and for everyone [19]. However, the LAN is also very common whereby, today, for example, every home Internet router has a switch or an access point that deploys a LAN, through which personal computers, printers, tablets, IoT devices, and portable phones can connect to the Internet [15].

As mentioned above, the digital world, with all its benefits, introduces significant privacy threats. For example, when frequently using applications, access is provided to sensitive data stored on our devices such the address book, photos, or user locations, which can be captured [20]. LANs are also vulnerable; specifically, due to their nature, WLANs are vulnerable to various types of attacks and introduce a significant threat to violate the user’s privacy [21]. Moreover, information that passes through the network, whether a LAN or a WAN, on wired or wireless connections, might be eavesdropped on with a variety of sniffing tools. One of the most prominent examples of these tools is Wireshark, a popular, free, and open-source software initially designed to analyze and improve network traffic, but which can also be used for malicious purposes. Therefore, theoretically, all communication transmitted on the network is vulnerable and might violate the user’s privacy. Other tools, such as an IP scanner, can scan the network by sending ping requests to all IP addresses in the segment, thereby revealing the network topology. Alternatively, they can scan all TCP and UDP ports to discover services on all network hosts [22].

There is an enormous number of studies on privacy on the Internet in many fields, e.g., e-health services (health services that are supported by digital technologies) [23]. However, when referring to the digital world in the context of privacy, the communication platform that is most commonly considered is the WAN, while the LAN is rarely addressed. For example, privacy issues are considered on online social networks like Facebook, Twitter, LinkedIn, Instagram, and TikTok, which indeed must be spread over the WAN to fulfill their purpose. This phenomenon is well reflected in the research field, e.g., seeking to place a price (in terms of privacy loss) for “free” services on the Internet like search engines or behavioral recommendations [4]. Another example is trying to study the privacy risks on the Internet that stem from sharing information on online social networks, including extreme scenarios such as identity theft [24]. However, as mentioned above, LANs are also very common; as a matter of fact, a LAN can be found in almost any home and any business or organization, and it definitely introduces privacy risks, which are inherent to this platform and, yet, neglected.

This research, unlike most others in the field of privacy, focuses on vulnerabilities that may introduce privacy threats in the LAN (rather than the WAN) environment. While LAN and WAN share standard components regarding both privacy and security, e.g., the decision-making strategy [25], they differ in the attention paid and the defense requirements. Given that the (a) LAN is prevalent, even more than the WAN; (b) privacy today is a major concern and referred to by both individuals and regulators; and (c) the scholarship almost does not address the issue of privacy in the LAN, researching the intersection between privacy and the LAN is of high importance. To distinguish the study from security issues, the vulnerabilities considered in this framework are those that do not involve breaches such virus infections, which may definitely result in a privacy violation but are not core privacy issues. Moreover, we consider a privacy vulnerability as one that does not require admin credentials to attain privacy-sensitive data, since the administrator must be trusted. To formalize the research question, first, LAN privacy vulnerabilities are defined. This definition encompasses the boundaries of the LAN (vs. WAN) and privacy (vs. security), as implemented by an inclusive criterion detailed in Section 3.1. Given these definitions, the research objective is to define a reproducible methodology for locating LAN privacy vulnerabilities, quantifying them beyond just indexing, and enabling their accommodation in a risk management model. The research empirical study maps these vulnerabilities, identifying privacy weaknesses in the LAN and estimating their incidence and magnitude.

## 2. Literature Review

The issue of privacy in LAN environments is illusive. Searching Google Scholar for the string “privacy on LAN” directs mainly to privacy on the wireless LAN. Most of the research seeks to increase awareness of privacy leaks that may occur when using public Wi-Fi networks, e.g., in wide places like airports or campuses with a large number of access points, where the layout can reveal the user’s location and movement in the space [26,27]. Other sources may be leakages of private details from a device or software that is connected to the Internet with a public Wi-Fi and may include computer name, domain name, web browser, and search engine regarding, for example, identity or financial or social privacy risks [28,29,30]. Another private details leakage concern may be caused by an interconnection of network elements, for example, connecting a HUB for sniffing private communication between two workstations [31]. A prevalent risk that was also noticed is related to the Internet of Things (IoT) technology.

Most organizations are equipped with a LAN; however, the attention to the workplace privacy aspect is usually from the employer’s point of view, who seeks to secure and keep the company’s privacy and its assets such as knowledge [32]. While these threats are certainly significant, they are all security issues. On the other hand, the employee’s privacy is less considered [33,34]. Even when the employees’ privacy is addressed, in many cases it relates to their physical privacy, e.g., working in an open space environment, rather than their digital privacy, e.g., working in the same LAN [35,36].

While the literature on LAN privacy is scant, some research do address this type of threat, e.g., printer issues. One of the most essential tools in business networks is the network printer, which enables sharing printing services among devices such as computers, tablets, and cellphones. The network printer is a source of one of the serious security problems [37]. For example, the protocol used for sending data to the printer is unencrypted; thus, it is hard to protect data that appears on paper. Namely, a user can easily send the print job to the wrong printer, and an unauthorized person can read the documents with personal data [38]. Some research has sought to fix this issue, e.g., develop a security model that ensures discretionary access control [39] or use encrypted data between the print server and the printers. Another vulnerability of network printers is its management website. By browsing the IP address of the printer, access to the management console of the printer may be gained.

A MAC address is a unique hardware identity of a Network Interface Card (NIC) in the LAN. By scanning the MAC address on the network, all hosts that belong to this network can be identified, introducing a significant privacy issue. To preserve privacy, modern operating systems have adopted the technique of using random MAC addresses to establish different Wi-Fi connections [40].

Another LAN privacy issue is related to the Domain Name System (DNS). Browsing a website or sending an email requires DNS services, which map Domain Names to IP addresses. Each DNS has one authoritative DNS server that keeps the domain record. The DNS is exposed to a variety of attacks and malicious activities on the Internet [41]. The DNS core service is applied on the WAN; however, due to the traffic volume, and in order to maintain high performance, there are recursive DNS which keep the last required DNS records in the cache, so that if they receive a request for the same record that is stored in the cache (at a timeframe of TTL), they do not need to send the request to the authoritative DNS and fetch it from the local cache [42,43]. While using a DNS cache on local servers may increase performance, it also increases the risk of DNS poisoning attacks, attacks that can change records in the DNS cache and redirect the browser to a spoof address with a fake website, which may violate privacy and security [44]. A DNS cache service may also present a privacy risk by revealing browsing history via its public access feature.

International business groups are increasingly using terminal systems as workstations. Currently, the security of terminal access systems is ensured by the robustness of the communication protocols between a terminal and a host [45]. Most of the research studies deal with access control in a cloud computing environment or the encryption of the cloud storage while maintaining high performance [46]. However, the privacy risk in the terminal system environment does not stem from the connection between the remote hosts and the server, but from the server itself. All the corporate users use the same server in different sessions, and though each user has their own profile folder, all users share storage and resources. Thus, when entering the task manager, all the tasks are exposed. The user’s privacy is pretty safe from external attacks but is exposed to threats from other users on the cloud server.

File sharing is one of the most popular services in the LAN. The ‘share folders’ function was a significant privacy vulnerability in the early Windows operating systems. Microsoft admitted that a wrong configuration could expose the computer to share all data with everyone on the LAN. Thus, Microsoft has changed the configuration to block all file-sharing access by default [47].

To reduce the response time of searching and opening emails on the Outlook application, Microsoft keeps an offline file that stores all the personal data locally (with the extension OST), where the user can set the time duration in which the data is stored. This file is part of the user’s profile data, like the Desktop, Documents, Downloads, and Pictures. The access to this folder is usually granted to the user only [48]. However, when using multiple terminal servers, the personal folder needs to roam between all servers, and one of Microsoft’s recommendations is to store all the users’ OST files in shared folders. This may enable other users to access these files [49].

Windows Push Notifications, presenting small popup windows with notifications like new emails that arrive, is part of the Windows operating system. The notifications window, which appears in the front, may contain sensitive private data, for example, the title of a message [50]. While this feature is valuable and therefore highly popular, it also introduces a significant privacy issue. This type of privacy risk is borderline regarding the current research because, on one hand, most of the notifications originate from the Internet, i.e., the WAN, but on the other hand, realizing this risk is local.

Managing attendance records in workplaces is a crucial task. The supporting technology of a time clock is developing, and today, the authentication mechanism may include RFID cards and biometrics such as facial recognition [51,52]. While applying biometric means is effective, it introduces security and privacy issues regarding the data that is stored. Some researchers are searching for solutions to secure the stored data of employees’ biometrics and facial identification [53,54]. However, one of the vulnerabilities that has not been sufficiently investigated is accessing the data on the time clock. This vulnerability is significant because, the time clock is usually installed on the LAN and other hosts in the same LAN may have access to this device through the IP. Ironically, this process of accessing the time clock is explained and documented in the manuals that are publicly available from the device manufacturer [55].

Some devices may store sensitive information, and are usually protected with a password mechanism. When the device is installed for the first time, or immediately after a factory default reset, the device password will be the default password that the manufacturer set, and since it can be found on the product website or the user manual, it is practically common knowledge [56]. For example, in October 2016, a wide cyber-attack was launched by using approximately 100,000 IoT devices like cameras, residential getaways, and baby monitors that were infected with the Mirai malware, targeting distributed denial of service (DDOS) attacks against sites like Twitter, Reddit, and The New York Times which were shut down for hours. Significant information stored on devices connected to the LAN may be protected from external attacks by blocking this access. However, since these devices are occasionally configured with default credentials, they are not protected against LAN access. Therefore, devices such as printers, DVR/NVR (recording videos in a digital format, usually from surveillance cameras), attendance systems, and access points may reveal sensitive data to unauthorized users connected to the same LAN. Other devices may stream sensitive information, e.g., closed-circuit televisions.

In recent years, mainly due to the COVID-19 pandemic, more and more employees are working remotely from their homes, accessing the company’s computing resources by using a VPN connection. However, some organizations also use popular free applications for remote access, like Any Desk or TeamViewer. These applications require a local client that is connected to an external server, enabling them to work behind the NAT (Network Address Translation) [57]. Applications like AnyDesk or TeamViewer require that both computers, the master and the client, be connected to the application server. However, other applications are able to connect directly to computers on the LAN, with no requirement for Internet access and without using the secured SSL protocol, e.g., VNC, Microsoft Remote Desktop, and Apache Guacamole. These remote applications use unique ports such as RDP (TCP 3389), PCoIP (UDP/TCP 4172), and others. Usually, the Firewall blocks such ports from access to the LAN (namely from the Internet). However, when accessing from within the LAN, such ports might be enabled and, thus, unprotected from internal users [58].

## 3. Methodology

The main goal of the research was to introduce a methodology that enables mapping vulnerabilities that may introduce privacy threats in a LAN environment. To this end, the research process methodology included the following primary phases: (A) defining inclusive criteria for vulnerabilities; (B) identifying and creating a theoretical list of vulnerabilities filtered by the criteria; and (C) testing a sample of LANs to identify practical vulnerabilities and evaluate them.

**Figure 1:**
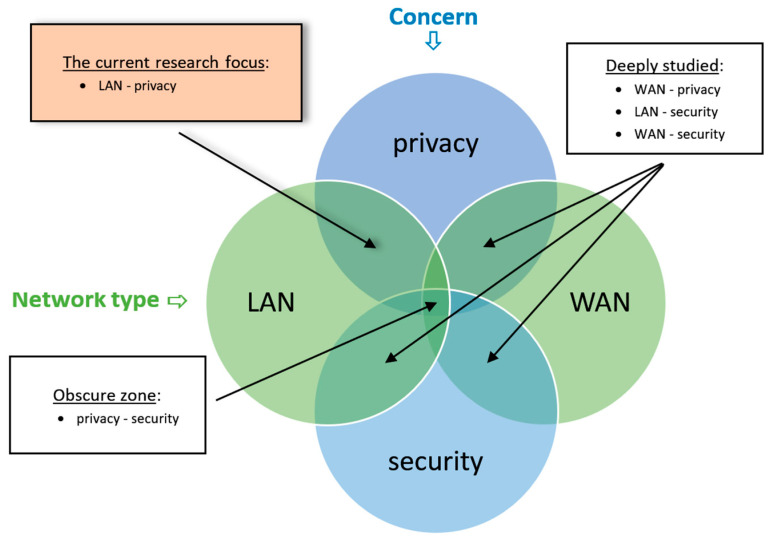
The LAN privacy model.

The LAN privacy model is therefore constructed on two main factors: (a) distinguishing LAN from WAN (or other networks); and (b) focusing on privacy issues rather than security. Getting ahead of the second factor is more obscure, while the first factor has relatively clear boundaries. The model is described in the Venn diagram depicted in Figure 1. As mentioned above, while privacy issues in the WAN, security issues in the WAN, and security issues in the LAN were thoroughly researched, the intersection between LANs and privacy issues was neglected; therefore, this is the current study’s focus.

To frame the LAN privacy vulnerabilities, a Failure Mode and Effects Analysis (FMEA) approach was adopted to evaluate the vulnerabilities. FMEA was initially developed by the U.S. military to mitigate failures. It is considered a common risk analysis tool. It covers two main concepts: (a) Failure mode, which describes, in the current research, the vulnerabilities and their ranking levels (the level of the threat as explained and detailed below); and (b) Effect analysis, which describes, in the current research, the privacy impact of the failure on the user [59]. The FMEA type that was applied in the current research is *process*, since the evaluation is not part of the design. Finally, mitigating the LAN privacy vulnerabilities can be driven directly from the identified vulnerabilities.

FMEA is not the only quantitative risk model that can be incorporated into the process of evaluating privacy vulnerabilities in a LAN. For example, the Hazard and Operability study (HAZOP), which was originally designed for the chemical industry, and later adopted in the software field, may also be applied here with some modifications [60]. HAZOP is applied to complex processes, and is based on discussions and interviews around guidewords, similarly to the process in the current study. Another example is the Probabilistic Risk Assessment (PRA) methodology, aimed at evaluating risks in complex engineering environments [61]. Similarly to the FMEA approach, PRA also considers two primary factors: the magnitude (severity) of the failure and the likelihood (probability) of the failure.

**Figure 2:**
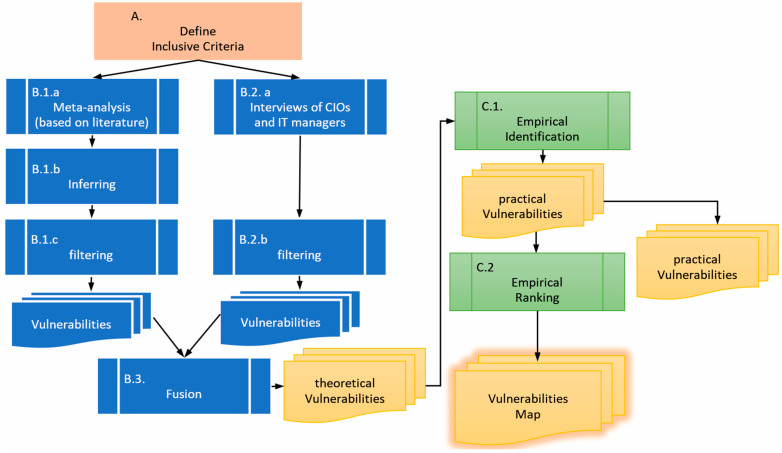
The methodological process.

The entire process is depicted in Figure 2. The process begins by defining the inclusive criteria to filter the identified vulnerabilities (phase A), as described in Section 3.1 below. In Phase B, vulnerabilities are identified through two means: inferred from the literature, which is usually focused on WAN or LAN security (B.1.a and B.1.b), and based on interviews with CIOs and IT managers (B.2.a), as described in Section 3.2 below. Then, both lists are filtered according to the criteria produced in phase A (B.1.c and B.2.b) and combined into a single list (B.3). The product of phase B is a list of theoretical vulnerabilities. In Phase C, the theoretical list of vulnerabilities is first empirically tested against real-world networks (C.1) to produce a practical list. Then, these vulnerabilities are evaluated and ranked (C.2), as described in Section 3.3 below, to produce a holistic vulnerability map.

### 3.1. The Vulnerability Inclusive Criteria

In order to identify relevant vulnerabilities in the LAN environment, four cumulative inclusive criteria were defined. A threat must meet all four criteria to be considered:

a. LAN environment:

The vulnerability can materialize in a LAN environment and does not require access to the WAN (the Internet). There are a few definitions that distinguish a LAN from a WAN; however, there is no significant disagreement regarding this classification. LAN is a computer network that connect local devices such as devices in the same room, or in the same building, or inside a single campus. In contrast, WANs connect devices that are relatively far apart [62]. The LAN definition in this regard also includes VLANs or LAN subnets. For example, accessing private data in a user’s profile working in the same LAN is considered a vulnerability; on the other hand, finding an open RDP (Remote Desktop) port by scanning public IP addresses is not considered a LAN environment vulnerability.

This research criterion focuses on the LAN platform, which is significant as described above. It does not necessarily narrow the type of vulnerability, since many vulnerabilities are present in both LAN and WAN, but it restricts the perimeter within which it may be considered.

b. Non-administrative rights:

The vulnerabilities can be materialized by a user who is granted non-administrative rights only. System administrators (sysadmins) and IT support typically own accounts with high privileges. These types of accounts and permissions are excluded. A non-administrative user can access data and resources that are owned by the user or shared, which may introduce relevant vulnerabilities. At the same time, this access, granted through administrative rights, will not be included. For example, accessing a private folder publicly shared by a user with a regular account with no administrative rights will be considered a risk. On the other hand, accessing a hidden share folder with admin user rights will not be considered in this research [63].

This criterion narrows down the research to relevant threats, since it may be assumed that an administrator who has almost free access to devices and data must be trusted in any case. Therefore, the criterion excludes a privacy violation in which a formally trusted person exploits their rights.

c. No hacking:

The vulnerabilities can materialize without the need of a hacking tool or the application of a hacking approach. Hacking tools are aids that assist hacking and are usually considered a form of cyber weapon [64]. Among the popular hacking tools are *packet sniffing*, *social engineering*, and *network scanning*. For example, fetching a user’s schedule from its Outlook calendar by accessing the PST file without the need to use software to gain access is considered in this research. On the other hand, using an SQL injection to capture details from a salary database will not be considered in this research.

This research criterion focuses on pure privacy rather than security issues. Together with the LAN criteria, it narrows down the vulnerability types to those that fall into the gap that has not been researched yet.

d. No password breach:

The vulnerabilities can materialize without guessing passwords, password brute force tools, or any other tool to breach passwords. For example, connecting to a HIKVISION NVR with the manufacturer’s default user and password (user: admin, password: hki12345) will be considered. On the other hand, this research will not consider accessing a device by using a hint like the user’s wife’s birthday date as a password.

This criterion is complementary to the ‘No hacking’ (c) criterion and might be considered its private case, serving the same purpose. The criterion is defined separately due to its popularity and because it does not entirely overlap with the ‘No hacking’ criterion.

### 3.2. Vulnerabilities’ Identification

Two sources were used to collect vulnerabilities according to the abovementioned criteria. The vulnerabilities collected from each source were merged into a single list. These sources are as follows:

- Interviews of CIOs (Chief Information Officer) and IT (Information Technology) experts on the subject of privacy risk, of which they are aware and/or that occur under their management. Since intuitively, when the term “privacy” is mentioned, the mind wanders to the Internet (the WAN). We expected that most of the vulnerabilities that they would raise would not fall within the criteria (especially criterion a); thus, the results needed to be filtered accordingly.
- Meta-analysis based on the relevant literature. Since the literature primarily does not deal directly with the current research problem, and in most cases security and privacy are confused, or “LAN” issues are actually Internet (WAN) issues, the criteria filter must be applied here as well. In the current research, the results are not averaged (like in most meta-analysis research) but cumulated.

The research was approved by the ethics committee of the Academic Institution (No. of Approval: AU-ENG-RH-20240728).

### 3.3. Vulnerabilities Ranking

Given the list of vulnerabilities that meet all four of the above criteria, in order to evaluate the level of each threat (caused by a vulnerability), two sets of ranks were used:

- Binary rank—for vulnerabilities that exist or not (without middle levels), a binary scale is defined. The ranking range is 0 if the vulnerability does not exist and 1 if it exists. For example, for the vulnerability of accessing the local DNS cache data, if they are not accessible, the vulnerability ranks is 0, and if they are accessible, the vulnerability ranks is 1.
- Multi-level rank—in vulnerabilities that have some level of threats, a multi-level discrete scale is defined. The ranking range may vary between different vulnerabilities according to the possible scenarios, where a 0 value is assigned if the vulnerability does not exist and values greater than 0 are assigned if it exists (the greater the value, the greater the vulnerability level). For example, for the vulnerability of accessing task manager data, if accessing the data is not possible the vulnerability rank is 0, if only open sessions are disclosed the vulnerability rank is 1, and if the entire list is disclosed the vulnerability rank is 2.

### 3.4. List of Vulnerabilities

The list of vulnerabilities that were identified and filtered according to the four criteria is detailed in Table 1. The table includes the vulnerability name, an explanation of the vulnerability, a description of the possible impact of this vulnerability on the user’s privacy, the materialization access platform, and the ranking range used to measure a specific vulnerability in a specific LAN. The materialization access platform indicates weather the vulnerability can be applied from any host in the LAN or from the local host which is the victim. For example, access to the printer management portal may be established from any host in the LAN, while access to a user’s profile may be established only from the ‘hot bed’ computer itself.

Among the 18 identified vulnerabilities, several demonstrate interdependencies or contextual correlations and may overlap. For instance, the detection of well-known ports (#3 Table 1) is only feasible following a comprehensive IP scan over a wired network (#1 Table 1) or IP scan over Wi-Fi (#2 Table 1). Certain vulnerabilities are conditional and manifest exclusively under specific circumstances. For example, popup notifications when screen is locked (#17 Table 1), unlocked computers (#8 Table 1), or the ability to boot the computer with an external source (#18 Table 1) required direct physical access to the machine. Similarly, vulnerabilities associated with printer log (#13 Table 1), video surveillance (#14 Table 1), or time clock information (#15 Table 1) are relevant solely within networks where such devices are deployed. Furthermore, actions such as accessing other user’s profile (#10 Table 1), local DNS data (#4 Table 1), password stored on shared workstations computers (#12 Table 1), or clipboard (#11 Table 1) are viable only in environments where multiple user accounts or profiles coexist on the same endpoint.

### 3.5. The Privacy Impact

Each vulnerability that is identified and ranked may have a different impact on the user’s privacy. For example, the IP scan over Wi-Fi vulnerability (#2 Table 1) may reveal the computer’s IP address, the MAC address of the network card, the computer’s name, and the operating system that is running, but without significant content. Therefore, its potential impact is low. On the other hand, the impact of access to other user profiles (#10 Table 1) may reveal all the user’s data content; thus, its impact is relatively very high. The impact level is actually the magnitude of the threat and the effects analysis of the FMEA model. In this research, the impact level is scaled between 1 and 5, where 1 represents the lowest impact and 5 represents the maximal impact.

The Overall Risk of a vulnerability is given by Equation (1).

(1) Overall Risk=Normalized Vulnerability·Impact level

## 4. The Empirical Study

### 4.1. Empirical Study Environment

For the empirical study, 13 different LANs were selected. The LAN’s organizational environments included factories, offices, home environments, coffee shops, and education institutes. The LANS also varied by the applied technologies, e.g., local LAN server, local remote server, cloud environment, and “Shared workstation”. In some organization, more than one LAN environment was found, and all of them were isolated and tested. Table 2 describes the LANs that were tested with a short description of the LAN architecture.

In the empirical study, all of the 18 vulnerabilities that were chosen were tested on each of the LANs. The tests were conducted in the same way on all of the networks, and the vulnerabilities were scored by the rank presented in Table 1. If a vulnerability was not relevant in a specific LAN, it was marked as N/A, e.g., IP scan over a wired network is not available on a Wi-Fi LAN.

### 4.2. Results

In light of the fact that not all vulnerabilities are measured on the same scale and with the same number of options, in order to yield a common and comparable scale, we normalized the results (Equation (2)) to produce a scale between 0 and 100 percent.

(2) $$Normalized\;Vulnerability\;Rank=\frac{Vulnerability\;Rank}{max\;(Ranking\;Range)}\cdot 100$$

For example, in the assessment of the vulnerability of the shared folders (#6 in Table 1), the ranking range is $\left\{0,1,2,3,4\right\}$; thus, network G, that received a vulnerability rank of 3, will have the Normalized Vulnerability Rank of $\frac{3}{4}\cdot 100=75\%$.

The final raw results are described in Table 3. Each row presents a specific vulnerability, while each column displays a specific network. If the vulnerability is irrelevant to the specific network, the cell is marked N/A. Therefore, each cell indicates the rank of a specific vulnerability in a specific network and includes the Normalized Vulnerability Rank (in percent) at the top, and the Vulnerability Rank (absolute value) in brackets at the bottom. For each LAN, the average per LAN, which appears at the bottom, is the average of all the Normalized Vulnerability Rank values for all applicable vulnerabilities in this LAN. For visualization purposes, the color of the cell is green for low-risk vulnerability (up to 25%), yellow for medium-risk (between 26% and 74%), and red for high-risk (over 75%). The right column indicates the average Normalized Vulnerability Rank across all LANs for each vulnerability. The bottom row describes the average Normalized Vulnerability Rank across all of the vulnerabilities for each specific LAN.

**Figure 3:**
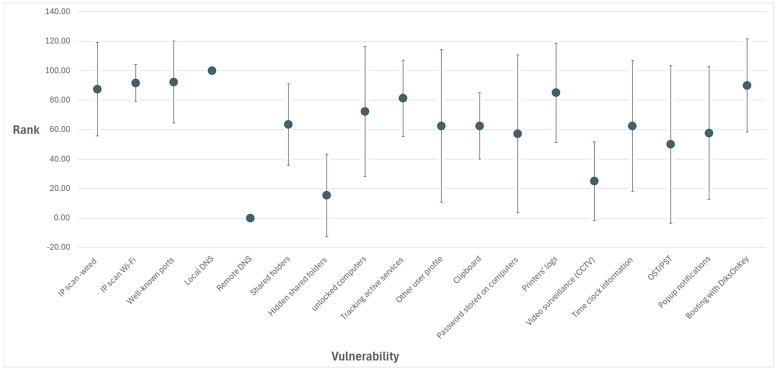
Visualization of the Vulnerabilities’ ranks across all LANs.

A summary of the vulnerability’s level values across all the LANS that were tested is provided in Table 4, and for a visual comparison, their general distribution is depicted in Figure 3. The middle marker represents the average value, and the whiskers represent the standard deviation values (the value is expressed by the size of the whisker and not by its ends).

As explained in the methodology section, while the vulnerabilities were identified and ranked, each may have a different impact on the user’s privacy. Therefore, the impact was evaluated in order to demonstrate how the overall risk can be mapped and quantified. It is important to note that the impact level, unlike the threat level, is a subjective value and has to be set by the users based on their privacy concerns. A summary of the average risks across all LANs is described in Table 5, which includes the list of all vulnerabilities with their Normalized Average Vulnerability, the Impact level for each vulnerability accompanied with an explanation, and the Average Overall Risk (across all LANs) which is attained by means of the multiplication described above.

**Figure 4:**
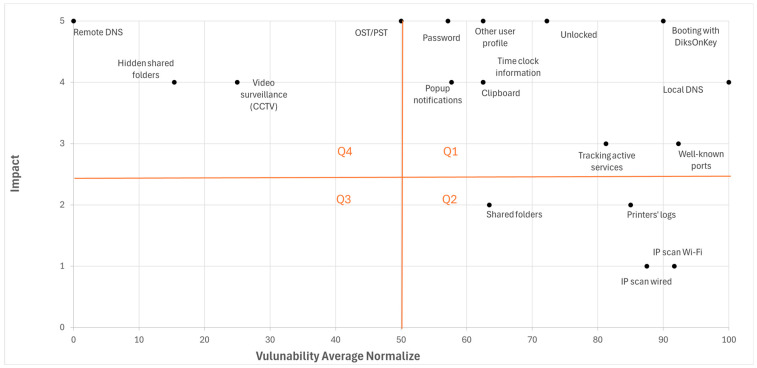
Map of the risks for each vulnerability.

Now, given the vulnerabilities’ levels (for each vulnerability in each LAN) and the impact level (for each vulnerability), a risk map may be drawn. The risk map of the empirical study results is depicted in Figure 4. In this illustrative map, the impact level of each vulnerability has the same value across all LANs; however, the methodology does not mandate this. The X-axis describes the Normalized Average Vulnerability, while the Y-axis describes the Impact level. Each point inside the map stands for a specific vulnerability in a specific LAN, indicating the abovementioned two values in a 2-dimensional space. The map was divided into four quarters. Naturally, vulnerabilities that are placed at the right upper quadrant (Q1) pose the highest risks, and those in the left lower quadrant (Q3) pose the lowest risks. Vulnerabilities in Q2 and Q4 present a medium risk with a high Normalized Average Vulnerability and a low Impact level or vice versa.

This research focuses on identifying vulnerability and assessing risk. However, to complete the frame and pave the way for further study, mitigation strategies are also proposed, as depicted in Table 6. The mitigations are divided into two groups: technological mitigation tools that the network administrator should consider adopting and installing, and awareness and behavioral mitigations that users should be educated on. The user aspect also seeks to break the asymmetry between attackers and denuders and involves decision-making [65].

## 5. Discussion

In this study we investigated the field of privacy vulnerabilities in the Local Area Network (LAN) environment. To focus on privacy and the LAN environment, four limiting criteria were applied to the research: (a) only LAN environments were observed; (b) administrative rights were not required to materialize the vulnerabilities; (c) no hacking actions were required to launch the vulnerabilities; and (d) no password breaching was involved. The vulnerabilities were identified by qualitative interviews with CIOs (Chief Information Officers) and IT (Information Technology) experts as well as by a meta-analysis of the relevant literature. While the analysis of previous studies is a legitimate methodology for identifying cyber risks [66], it makes a relatively small contribution in the current case, as this topic has not been widely researched. In total, 18 vulnerabilities were found. Following the first stage of identifying the vulnerabilities, an empirical study was conducted, in which all 18 vulnerabilities were evaluated quantitively in 10 different corporations on 13 different LANs. From the results of this study, we can conclude that almost all of the identified vulnerabilities exist in at least one LAN, and only one vulnerability was not found on any LAN. More than 50 percent of the vulnerabilities pose the highest risks (Normalized Average Vulnerability as well as a high Impact level, and the others pose a medium risk.

Privacy awareness has been increasing together with the growing number of Internet connections [67]. Consequently, studies on privacy have investigated the Internet user aspects, e.g., the ability to gather information about social network users [68]. While the discussion on privacy has focused mainly, and perhaps even solely, on the Wide Area Network (WAN) environment (with the Internet as its major application), almost every office, house, public facility, and any other place with an Internet connection has a LAN environment that applies a router as a gateway to the Internet, and sometimes local applications are installed on the LAN itself. Practically, in homes and small businesses, the ‘box’ which is popularly called a “router” also includes a switch and an access point that deploy a wired/wireless LAN between the local devices. In larger organizations, the router may be a dedicated device, with a switch that deploys the LAN. Sometimes, there is even a LAN without a router, e.g., highly secured sites that are not connected to the Internet.

Most jobs involve some type of use of computers, e.g., sending emails, using office applications, and operating specific software. Employers tend to monitor the employees to ensure that the work is performed efficiently and sometimes, to prevent information leakage [32]. Although employees are aware of their employer’s ability to access private data such as emails, documents, and activities that are implemented on the employer’s computer, they continue with such activity, while sometimes claiming that “I have nothing to hide” [69]. Therefore, the privacy issue on the LAN environment is significant, especially given the exposure to other users on the LAN to whom the user is not a subordinate to, and the awareness of privacy violation in these cases is limited or does not exist at all.

Despite the immense distribution of the LAN environment, very few studies have dealt with the issue of privacy on a LAN, and their primary concern has been the physical environment, e.g., the employee’s potential privacy violation via cameras [34], or biometric physical access control [70], or revealing a user’s location by measuring the broadcast power on Wi-Fi antennas that are spread throughout a campus [28]. The centrality of the privacy issue, the large-scale distribution of LANs, and the limited number of studies on the topic of privacy in LAN environments introduce a paradox. This phenomenon is empowered by the empirical study results which indicate that significant privacy threats in LAN environments are not theoretical, but actually exist. Therefore, this research makes a significant contribution to both the academic and industry fields.

This research identified and tested 18 vulnerabilities. However, the question of ‘what privacy vulnerabilities may exist in a LAN environment’ is an open one, and consequently, there is no certainty that all vulnerabilities were indeed identified. Furthermore, because the technology is highly dynamic, e.g., changes and upgrades to the operating system, to the network, and to the applications, the risks of these vulnerabilities may decrease or increase, and new vulnerabilities may appear. For example, popup notifications when the screen is locked (#17 Table 1) can disclose the entire message. However, for example, a WhatsApp update reduced the risk of this vulnerability by disclosing only a title in the popup notification. On the other hand, adopting a new technology such as Near-field communication (NFC) can introduce new vulnerabilities. For instance, when an NFC device like a smartphone is “borrowed” for a moment, internal information may be disclosed by means of NFC printing [71]. Another limitation of this research is that interviews with CIOs and IT experts include subjective aspects which may include bias due to the inconvenience of reporting vulnerabilities in the environment that are under their responsibility. However, given that only a few experts were interviewed, and given that each vulnerability that was identified was tested on all LANs (also on those where the CIO did not report it), in other words, all the identified vulnerabilities and all the LANs were crossed-tested; hence, this limitation is mitigated. Future research may extend the number of LANs examined in order to discover more vulnerabilities (if they exist) and to increase the accuracy of the vulnerabilities’ rank evaluation. In addition, given the abovementioned dynamic characteristics of the technological environment, the study should be repeated periodically. This action is common in the IT industry, and is known as a risk survey [72]. Lastly, while this research identifies privacy issues in the LAN environment, formally, the methodology does not provide solutions. Some solutions may be a straightforward derivate of the problem definition, for example, raising awareness of the vulnerabilities on the LAN and providing training to educate users can prevent some of these risks. Future studies may also propose methodologies to provide further solutions to this neglected problem.

Privacy is a major concern and lies in the heart of public, academic, and industrial discussion. The threats and solutions in the WAN environment should be integrated with those in the LAN environment to form a holistic scope of this field.

## Figures and Tables

**Table 1:** The list of vulnerabilities.

	Vulnerability	Explanation	Privacy Implications	Ranking Range	Materialization Access Platform
1	IP scan over a wired network	A simple scan of the LAN using an available utility can reveal devices that are currently connected. The information may include the IP address, computer name, device type, MAC address, and device status. Scanning the subnet mask can reveal devices currently connected to other VLANs.	It is possible to deduce if a user is connected and available. In other words, if an individual is absent or is working.	{0, 1, 2, 3, 4} 0 no threat 1 only the IP address is revealed 2 IP address and device name are revealed 3 the entire host’s details are revealed 4 hosts from other VLANs are also revealed	Any host in the LAN
2	IP scan over Wi-Fi	A simple scan of the Wi-Fi network using an available utility can reveal currently connected devices, including private devices such as smartphones and notebooks. The information may include IP address, computer name, device type, MAC address, and device status.	It is possible to deduce if a user is connected and available. If MAC randomization is applied, the user can still be tracked within a specific network, but the information cannot be crossed between networks. Moreover, in a wide area Wi-Fi network that includes access points, the location of the user may be disclosed.	{0, 1, 2, 3, 4} 0 no threat 1 only IP address is exposed 2 IP address and device name are exposed 3 the entire host’s details are exposed 4 hosts from other VLANs are also exposed	Any host in the LAN
3	Well-known ports	By mapping the open ports on the LAN, it is possible to identify which applications and services are active on a specific device.	It is possible to disclose activities that the user is conducting, e.g., file sharing, remote access, and printing. (Moreover, knowing the open ports may be useful to carry out cyber-attacks that may compromise privacy; but this does not fall within the inclusion criteria of this research).	{0, 1} 0 no threat 1 threat exists	Any host in the LAN
4	Local DNS data	By accessing the local DNS data, it is possible to intercept DNS request contact. This attack can be launched only from the specific PC.	When a user is browsing the Internet, the DNS requests may be of interest to another user who has access to this computer, thus disclosing the first user’s browsing history.	{0, 1} 0 no threat 1 threat exists	The local host
5	Remote DNS data	By accessing the DNS server data, namely the DC DNS or the router DNS, it is possible to intercept DNS request contact. This attack can be launched in all the LANs.	When all the users browse the Internet, the DNS requests may be of interest to another user who connects to the same LAN, thus disclosing all the users’ browsing history on this LAN.	{0, 1} 0 no threat 1 threat exists	Any host in the LAN
6	Shared folders	By using a network discovery tool (which is publicly available even as a built-in Windows feature), it is possible to discover all the devices on the LAN with open share folders. The user actively initiates the sharing (the information includes the computers’ names and sharing names). Thus, unless permissions are limited, there is access to these folders’ content.	It is possible to access shared files which may contain sensitive data.	{0, 1, 2, 3, 4} 0 no threat 1 list of folders and/or files is enabled 2 folder and files read (open files) is enabled. 3 folder and files write/edit is enabled. 4 the user is granted full control permission	Any host in the LAN
7	Hidden shared folders	Accessing Microsoft default hidden shared folders that were created by the operating system as part of the system architecture (e.g., \C$, \ADMIN$, \IPC$) enables the user to access these folders’ content and even content on the entire disk.	Sensitive data on shared folders or even on the entire disk may be disclosed.	{0, 1, 2, 3, 4} 0 no threat 1 list of folders and/or files is enabled 2 folder and files read (open files) is enabled. 3 folder and files write/edit is enabled. 4 the user is granted full control	Any host in the LAN
8	Unlocked computers	When a computer is not locked, other users have access, at times by only a slight move of the mouse (in many cases, users rely on the auto-lock mechanism, in which case, until the auto-lock activates vulnerability exists).	It is possible to access all the data that the last user had permission to access.	{0, 1, 2} 0 no threat 1 the screen is black, but the computer is not password protected when the mouse is slightly moved 2 the screen is on.	The local host
9	Tracking active users’ Windows services	Opening the Task Manager on a local PC or a terminal server allows all of the applications and services currently running on the PC to be viewed (even those used by other users).	The list of all applications and services that currently run (used) can be disclosed.	{0, 1, 2} 0 no threat 1 only the open session is disclosed 2 the entire list is disclosed	The local host
10	Other user’s profile	Opening the user’s folder on a local disk displays the list of all users that are logged on to the PC. Opening the user’s folder enables access to all the user’s profiles.	The user profile includes Desktop, My Documents, pictures and more.	{0, 1} 0 no threat 1 threat exists	The local host
11	Clipboard	By pasting from the clipboard, the last object copied can be disclosed. If the Windows clipboard history is enabled, a list of the last copied objects can be disclosed. The clipboard may preserve its contents even after the user is changed.	The clipboard can contain sensitive text, pictures, passwords, and other objects.	{0, 1, 2} 0 no threat 1 clipboard data is available if the user is still logged on 2 clipboard data is available even after the user is changed	The local host
12	Password stored on shared workstations computers	Accessing the password manager on Google Chrome or Microsoft Edge discloses all the passwords stored in the profile (other third-party software may also be vulnerable).	Sensitive information, for example, on private sections of websites, can be disclosed.	{0, 1} 0 no threat 1 threat exists	The local host
13	Printer log	By browsing the printer IP address, it is possible to access the printer management portal and view the print job log.	The log may contain sensitive data such as the name of the user and names of files that were printed.	{0, 1, 2} 0 no threat 1 the default password enables access 2 no password is needed	Any host in the LAN
14	Video surveillance (CCTV)	A LAN mapping can discover NVR/DVR devices that are currently connected, and the video stream and even the recording may be accessed by means of the default password.	Live broadcasts and recordings may contain sensitive data.	{0, 1, 2} 0 no threat 1 the default password enables access 2 no password is needed	Any host in the LAN
15	Time clock information	A LAN mapping can expose the time clock that is currently connected, and by using a free utility that can be downloaded from the manufacturer’s website, it is possible to access the clock’s database.	The time clock contains sensitive data about the present and past workers.	{0, 1, 2} 0 no threat 1 the default password enables access 2 no password is needed	Any host in the LAN
16	OST/PST (Microsoft outlook data files)	Searching for OST or PST file extensions on the local disk or on shared folders can disclose other users’ email data.	Personal email data may contain sensitive data, including email contacts, address book, and attached files.	{0, 1} 0 no threat 1 threat exists	The local host
17	Popup notifications when screen locked	When the computer is locked notifications can still pop up on the screen.	Personal data such as massages can be disclosed.	{0, 1, 2} 0 no threat 1 only title is disclosed 2 the entire message is exposed	The local host
18	Booting the computer with an external source	Booting the computer from an external source such as a DiskOnKey or a CD, can disclose the entire data that is stored on the hard disk (unless encrypted).	The hard disk may contain sensitive data.	{0, 1, 2} 0 no threat 1 it is possible to boot from an external source, but additional actions are required (e.g., driver installation) 2 it is possible to boot from an external source without any additional requirements	The local host

**Table 2:** List of the LANs’ environments in the empirical study.

Organization Number	Organization’s LAN	Organization Type	LAN Environment Architecture
A	1	Educational institution	A lecturer’s computer is installed in the classroom and students’ computers are installed in the Lab classes. All of these computers are connected to a domain. Logging into a computer is via a local profile.
A	2	Educational institution	There is free Wi-Fi throughout the campus.
B	1	Nuclear Pharmacy	Workstations are shared between users of the organization’s QC (Quality Control) stations and the production station. Every user connects to their own AD (Active Directory—Microsoft’s proprietary directory service that enables management pf permissions and access to network resources). All workstations have more than one user profile and are connected by a wired LAN.
B	2	Nuclear Pharmacy	Workstations are installed in the product and QC areas. Some of the workstations’ login process is personal, while others are based on generic user names.
B	3	Nuclear Pharmacy	There are Wi-Fi access points with visible SSIDs (Service Set Identifier—the name of the Wi-Fi network) and passwords throughout the factory.
C		Factory	There is a DC (Domain Controller—a server that is responsible for managing the network and identity security requests) server with shared folders and two ERP servers (two different software). All of the workstations are connected to an AD, and the users access the ERP application by means of personal user accounts.
D		Hotel chain	Servers are hosted in Google farm. The DC, application servers, and four Terminal servers share the same LAN with a roaming profile (the user profile is stored on a shared folder and by applying roaming, this information is shared on the four servers). Each Hotel is connected to the servers by a site2site VPN, where most stations are part of the domain. Some users have personal accounts, others like house-keeping and reception use functional accounts.
E		Factory	A local network with a DC, ERP server, and a Terminal server. All users are connected through a local Workstation to an AD and run a Terminal server client from the desktop. Only the engineering department works locally with a CAD (Computer Aided Design) software.
F		International trade agency (import and export)	A local network with a shared NAS and P2P architecture. Each user has their own profile, while some of the PCs contain profiles of more than one user. Some users have a profile on more than one PC.
G		Mechanical equipment trading (wholesale and retail)	There is a site2site connection between the head office and two branch offices. DC and ERP are available for all users, where remote users are connected via the terminal server and head office users are connected via a LAN.
H		Home environment	Home LAN with a few workstations (each with its own profile), laptops (belonging to the homeowner’s workplace), cell phones, a network printer, and IoT cameras.
I		Public Wi-Fi in a coffee shop	A coffee shop with free Wi-Fi, accessible by customers with laptops and cell phones.
J		Educational institution	A LAN that includes classroom PCs in an academic campus, which are mainly used for presentations. The computers are connected by wire, have a free shared profile, and have no domain definitions.

**Table 3:** Detailed vulnerability ranks for each LAN.

Vulnerability	Ranking Range	LAN Environment													Normalized Average per Vulnerability
		A1	A2	B1	B2	B3	C	D	E	F	G	H	I	J	
IP scan-wired	{0, 1, 2, 3, 4}	100 (4)	N/A	100 (4)	0 (0)	N/A	100 (4)	100 (4)	75 (3)	100 (4)	100 (4)	100 (4)	N/A	100 (4)	88%
IP scan Wi-Fi	{0, 1, 2, 3, 4}	N/A	100 (4)	N/A	N/A	75 (3)	100 (4)	75 (3)	75 (3)	100 (4)	100 (4)	100 (4)	100 (4)	N/A	92%
Well-known ports	{0, 1}	100 (1)	100 (1)	100 (1)	0 (0)	100 (1)	100 (1)	100 (1)	100 (1)	100 (1)	100 (1)	100 (1)	100 (1)	100 (1)	92%
Local DNS	{0, 1}	100 (1)	N/A	100 (1)	100 (1)	N/A	100 (1)	100 (1)	100 (1)	100 (1)	100 (1)	100 (1)	N/A	100 (1)	100%
Remote DNS	{0,1}	0 (0)	0 (0)	0 (0)	0 (0)	0 (0)	0 (0)	0 (0)	0 (0)	0 (0)	0 (0)	0 (0)	0 (0)	0 (0)	0%
Shared folders	{0, 1, 2, 3, 4}	75 (3)	100 (4)	25 (1)	0 (0)	50 (2)	75 (3)	75 (3)	75 (3)	75 (3)	75 (3)	75 (3)	50 (2)	50 (2)	63%
Hidden shared folders	{0, 1, 2, 3, 4}	25 (1)	25 (1)	0 (0)	0 (0)	0 (0)	0 (0)	0 (0)	25 (1)	100 (4)	25 (1)	0 (0)	0 (0)	0 (0)	15%
Unlocked computers	{0, 1, 2}	100 (2)	N/A	0 (0)	0 (0)	N/A	100 (2)	100 (2)	50 (1)	100 (2)	100 (2)	100 (2)	N/A	N/A	72%
Tracking active services	{0, 1, 2}	100 (2)	N/A	100 (2)	50 (1)	N/A	N/A	50 (1)	100 (2)	100 (2)	50 (1)	N/A	N/A	100 (2)	81%
Other user profiles	{0, 1}	0 (0)	N/A	100 (1)	N/A	N/A	N/A	0 (0)	100 (1)	100 (1)	0 (0)	100 (1)	N/A	100 (1)	63%
Clipboard	{0, 1, 2}	50 (1)	50 (1)	50 (1)	50 (1)	50 (1)	50 (1)	50 (1)	50 (1)	100 (2)	50 (1)	100 (2)	N/A	100 (2)	63%
Password stored on computers	{0, 1}	N/A	N/A	0 (0)	0 (0)	N/A	N/A	100 (1)	0 (0)	N/A	100 (1)	100 (1)	N/A	100 (1)	85%
Printers’ logs	{0, 1, 2}	0 (0)	N/A	100 (2)	100 (2)	100 (2)	100 (2)	100 (2)	100 (2)	100 (2)	100 (2)	50 (1)	N/A	N/A	85%
Video surveillance (CCTV)	{0, 1, 2}	N/A	N/A	0 (0)	0 (0)	0 (0)	50 (1)	0 (0)	50 (1)	N/A	50 (1)	50 (1)	N/A	N/A	25%
Time clock information	{0,1,2}	N/A	N/A	50 (1)	0 (0)	0 (0)	100 (2)	50 (1)	100 (2)	100 (2)	100 (2)	N/A	N/A	N/A	63%
OST/PST	{0, 1}	N/A	N/A	100 (1)	0 (0)	0 (0)	100 (1)	100 (1)	0 (0)	100 (1)	0 (0)	N/A	N/A	N/A	50%
Popup notifications	{0, 1, 2}	0 (0)	0 (0)	100 (2)	0 (0)	100 (2)	100 (2)	100 (2)	100 (2)	100 (2)	50 (1)	50 (1)	50 (1)	0 (0)	58%
Booting with DiskOnKey	{0, 1, 2}	100 (2)	N/A	100 (2)	0 (0)	N/A	100 (2)	100 (2)	100 (2)	100 (2)	100 (2)	100 (2)	N/A	100 (2)	90%
Average per LAN		54%	53%	61%	19%	44%	79%	67%	68%	93%	67%	75%	50%	68%	62%

**Table 4:** Vulnerabilities’ ranks across all LANs.

Vulnerability	Min Value	Max Value	Average	Standard Deviation	Number of N/A
IP scan over wired network.	0	100	87.5	31.73	3
IP scan over Wi-Fi	75	100	91.67	12.50	4
Well-known ports	0	100	92.31	27.74	0
Local DNS data	100	100	100.00	0	3
Remote DNS data	0	0	0	0	0
Shared folders	0	100	63.46	27.74	0
Hidden shared folders	0	100	15.38	28.02	0
Unlocked computers	0	100	72.22	44.10	4
Tracking active users’ Windows services	50	100	81.25	25.88	5
Other user profiles	0	100	62.50	51.75	5
Clipboard	50	100	62.50	22.61	1
Password stored on shared computers	0	100	57.14	53.45	6
Printers’ logs	0	100	85.00	33.75	3
Video surveillance (CCTV)	0	50	25.00	26.73	5
Time clock information	0	100	62.50	44.32	5
OST/PST (Microsoft Outlook data files)	0	100	50.00	53.45	5
Popup notifications when screen locked	0	100	57.69	44.94	0
Booting the computer with DiskOnKey	0	100	90.00	31.62	3

**Table 5:** The overall risks.

Vulnerability	Normalized Average Vulnerability (%)	Impact Level ({1,2,3,4,5})	Impact Level Explanation	Average Overall Risk (0–5)
IP scan over wired network	87.50	1	The information is limited to the computer’s IP address and name only.	0.88
IP scan over Wi-Fi	91.67	1	The information is limited to the computer’s IP address and name only.	0.92
Well-known ports	92.31	3	Software that are used by the user may be exposed, but not the content.	2.77
Local DNS data	100.00	4	Browsing history of the local computer may be exposed.	4.00
Remote DNS data	0	5	Browing history of the entire LAN may be exposed.	0
Shared folders	63.48	2	Shared data, which is usually not private, may be exposed.	1.27
Hidden shared folders	15.38	4	Shared data, which might be private, may be exposed.	0.61
Unlocked computers	72.22	5	Access to an unlocked computer may compromise all users’ data.	3.61
Tracking active users’ Windows services	81.25	3	Services and software that are used on the PC may be exposed, but not their content.	2.44
Other user profiles	62.50	5	All users’ profiles, including the users’ documents, may be exposed.	3.13
Clipboard	62.50	4	The clipboard history of other users that used the same PC may be exposed.	2.50
Password stored on shared workstations computers	57.14	5	Users’ passwords may be compromised.	2.86
Printers’ logs	85.00	2	Printers’ job history may be exposed, but not the printed content.	1.70
Video surveillance (CCTV)	25.00	4	Videos of the environment (live or recordings) may be exposed.	1
Time clock information	62.50	4	Attendance of employees may be exposed.	2.50
OST/PST (Microsoft outlook data files)	50.00	5	The outlook database which includes emails, calendar, contacts, etc., may be disclosed.	2.50
Popup notifications when screen locked	57.69	4	Visible popup notification messages may be exposed to all users.	2.31
Booting the computer with DiskOnKey	90.00	5	All the disk content may be disclosed.	4.50

**Table 6:** Mitigation Strategies.

	Vulnerability	Technological Tools (Actions that Are Not Taken by the User)	Awareness and Behavioral (Actions that Are Taken by the User)
1	IP scan over a wired network.	Block the ICMP protocol, and use an IDS system to detect scanning on the LAN.	-
2	IP scan over Wi-Fi	Block the ICMP protocol, and use an IDS system to detect scanning on the LAN.	-
3	Well-known ports	Use an IDS system that detects port scanning over the LAN.	
4	Local DNS data	Clear local flush DNS on every login.	
5	Remote DNS data	DNS security hardening.	
6	Shared folders	Scanning the LAN for insecure shared.	Do not open a shared folder with weak permissions.
7	Hidden shared folders	A software agent that ensures that no user possesses privileges exceeding the self-operational needs.	
8	Unlocked computers	Enforce the group policy of the lock screen on unused PCs	Lock your screen when you leave your workplace.
9	Tracking active users’ Windows services	Ensuring that no users possess privileges exceeding their operational needs.	
10	Other user’s profile	A utility that checks that users do not have local administrator privileges.	
11	Clipboard	Create a script that cleans the clipboard on logout.	Keep the clipboard clean when leaving the workstation.
12	Password stored on shared workstations computers	Disable the ‘Save Password’ option on shared workstations.	Do not save passwords on shared workstations.
13	Printer log	If the log printer access is not public, the default password must be changed.	
14	Video surveillance (CCTV)	Video system default password must be changed. The CCTV system should be on a separate VLAN with a limited access.	
15	Time clock information	Time clock default password must be changed. Time clock system should be on a separate VLAN with limited access.	
16	OST/PST (Microsoft outlook data files)	Use encrypted OST/PST file. Keep files on home directory with limited access	
17	Popup notifications when screen locked	Keep the system updated.	Close application when leaving the workplace.
18	Booting the computer with an external source	Encrypt the disk. Disable access to the PC. Use a BIOS password.	

## Data Availability

The data presented in this study are available anonymously on request from the corresponding author.
